# Ultrasound Diagnosis of Hamstring Muscle Complex Injuries Focus on Originate Tendon Structure—Male University Rugby Players

**DOI:** 10.3390/diagnostics15010054

**Published:** 2024-12-28

**Authors:** Makoto Wada, Tsukasa Kumai, Takumi Okunuki, Takeshi Sugimoto, Kotaro Ishizuka, Yasuhito Tanaka

**Affiliations:** 1 Department of Orthopedic Surgery, Wada Orthopedic Clinic, Osaka 5730163, Japan; 2 Graduate School of Sport Sciences, Waseda University, Saitama 3591192, Japan; t.okunuki@akane.waseda.jp; 3 Faculty of Sport Sciences, Waseda University, Saitama 3591192, Japan; 4 Research Organization of Science and Technology, Ritsumeikan University, Shiga 5258577, Japan; 5 Department of Orthopedic Surgery, Osaka Global Orthopedic Hospital, Osaka 5360008, Japan; sugimoto.tks.db@icloud.com; 6 Department of Orthopedic Surgery, Sports Medicine Center, Urasoe General Hospital, Okinawa 9012102, Japan; bonjour.machao1001@gmail.com; 7 Department of Orthopedic Surgery, Nara medical university, Nara 6348521, Japan; yatanaka@naramed-u.ac.jp

**Keywords:** hamstring, ultrasound, muscle injury, muscle strain, injury location, classification

## Abstract

**Objective:** With the remarkable advances in diagnostic ultrasound equipment, there is a growing need for ultrasound diagnosis of muscle and soft tissue injuries in sports injuries. Among these, hamstring strains are often difficult to treat and require early and accurate diagnosis. Injuries to the proximal part of the hamstring often take a long time to heal. For this reason, the diagnosis of proximal hamstring injuries is extremely important. The structure of the origin tendon is characteristic, and it is a complex in which the semitendinosus muscle (ST) of the medial hamstring and the long head of the biceps femoris muscle (BFLH) of the lateral hamstring share a conjoint tendon (CT). On the other hand, the semimembranosus muscle (SM) attaches to the ischial tuberosity independently. In this study, we created a classification of injury sites focusing on the origin tendon, and investigated the distribution of injury location, relationship to the player’s position, and the detection rate of ultrasound diagnosis. **Material and Methods:** We used ultrasound and MRI to diagnose 52 university men’s rugby players who had suffered a hamstring strain for the first time and investigated the distribution of the injured areas. We performed an ultrasound scan as the initial diagnosis and used MRI as a final diagnostic tool. A classification focusing on the origin of the muscle was created. First of all, it was divided into two types: the BFLH-ST complex type, which originates in the CT, and the SM type, which originates in the SM tendon. We also classified BFLH-ST complex damage, including CT damage, as Type I, a BFLH injury without CT injury as Type II, and a ST injury without CT injury as Type III. We then investigated the distribution of the injury location. The degree of ultrasound detection in each injury type was evaluated in three grades. The frequency of BFLH complex and SM injuries was investigated in players who played the forward (FW) and back (BK) positions. **Results:** The distribution was 40 limbs (77%) for BFLH-ST complex injury type and 12 limbs (23%) for SM injury type. In the BFLH complex type,19 limbs which met the Type I classification criteria for CT tear, 19 limbs met the Type II, and 2 limbs met the Type III. FWs had a higher incidence of SM injuries and BKs had a higher incidence of BFLH-ST complex injuries. With regard to the detection of muscle injuries via ultrasound, a high rate of detection was possible, except for a slight injury to the myofascial junction of the BFLH. **Discussion:** In terms of the distribution, the BFLH-ST complex, which shares the same origin tendon (i.e. CT), had a higher frequency of muscle tears than the SM. In addition, CT junction injuries occurred frequently in Type II as well as Type I (=CT injury). One possible cause is that the CT is subject to concentrated traction stress from both the medial and lateral hamstrings. With ultrasound, the detection rate of muscle damage around the BFLH-ST complex and SM originating tendon was high, suggesting that it is useful as an initial diagnosis. From this, it can be said that ultrasound is also useful for primary evaluation of “proximal hamstring injury”, which is prone to severe and should be given a final diagnosis using MRI. **Conclusions:** We created a classification system focusing on the originating tendons and clarified their incidence rates. In this study, ultrasound was found to be useful in the detection of originating tendon injuries. We also identified the characteristic sonographic findings of each type.

## 1. Introduction

Of all sports injuries, muscle injuries are the most common [1], and in particular, cases of hamstring muscle complex (HMC) injuries that recur or become serious are often discussed [2,3,4]. Ultrasound and MRI scans are useful for evaluating muscle damage [5,6], but there are many MRI classifications [6] and evaluations, such as the BAMIC classification [7]. The proximal tendon of the HMC has a very distinctive anatomical structure [3,8,9]. The semitendinosus muscle (ST) and the long head of the biceps femoris (BFLH) share a conjoint tendon (CT) [10], which attaches to the ischial tuberosity. On the other hand, the semimembranosus (SM) muscle attaches to the ischial tuberosity on its own. No classification to date has taken into account the characteristic structure of this proximal tendon’s origins. In research on muscle activity using electromyography, activation of the BF was significantly greater during the early stance phase than the late stance phase (*p* < 0.01); the medial hamstring (MH) had significantly greater EMG activation during the late stance (*p* < 0.05) and mid-swing (*p* < 0.01) phases [11]. As you can see, tendons with different functions share the same origin, and in CT, the load is applied in all phases of running. Injuries to this proximal area often require a long time to recover from and may even require a surgical indication [5,9,12]. We focused on the proximal tendon structure of the HMC and classified it into two types: one in which the BFLH and ST originate from the common part of the proximal tendon, and the other in which the SM originates from the semimembranosus tendon. We investigated the site of injury and clarified the frequency. Then, we divided the players into two groups, forwards and backs, and investigated the difference in the distribution of muscle tears between the BF-ST complex and the SM. We performed ultrasound and MRI on all cases, at each injury site, the ultrasound detectability and the characteristic ultrasound image of the proximal tendon injury are shown.

## 2. Materials and Methods

This is a prospective study of 52 male university rugby players who were diagnosed with HMC injury within two weeks of their first injury at our hospital outpatient clinic between September 2020 and September 2024.

**Figure 1:**
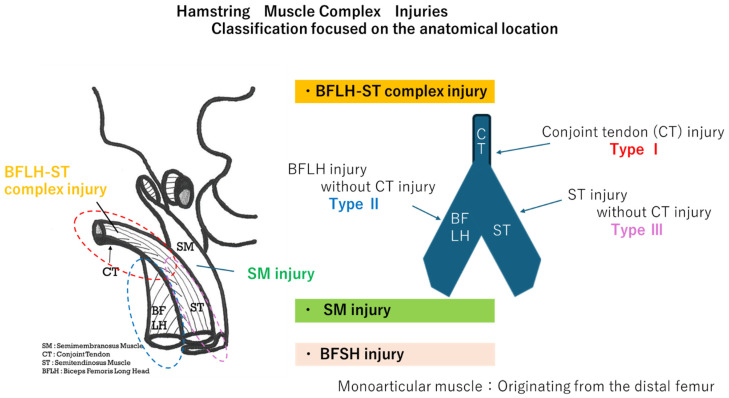
Injury site classification.

All subjects were assessed using sonography and MRI. After the ultrasound examination, an MRI examination was performed, and the final diagnosis was made based on the MRI examination results. Cases of recurrence and injury due to direct external force were excluded. When two injuries were present on imaging, a detailed history was taken and compared with the present condition to strictly determine which was the first injury. If it was not the first injury, it was then excluded. We classified the hamstrings based on their tendon origins. For the locational classification, the BFLH and ST sharing a proximal conjoint tendon were designated the BFLH-ST complex (Figure 1). First, we divided hamstring muscle complex injuries (HMCIs) into monoarticular muscles and biarticular muscles. Then, we focused on the origin tendon of the biarticular muscles and classified them into the BFLH-ST complex and the SM. In the BFLH-ST complex, we defined Type I as an injury in which the CT was damaged and it was thought that both the BFLH and ST were damaged. Those with no CT injury and thought to have muscle damage on one side only were classified as Type II (BFLH side) or Type III (ST side). We divided players into forwards (FWs; positions No.①–⑧) and backs (BKs; positions No.⑨–⑮) and investigated the frequency of muscle injuries to the BF-ST complex or the SM.

Ultrasound diagnosis was performed by an orthopedic surgeon who had 10 years of experience interpreting ultrasound images. There were two doctors who read the MRI images; the radiologist had 35 years of experience and, the orthopedic surgeon had 30 years of experience.

The ultrasound devices were Aplio i700 (Canon Medical Systems, Otawara, Japan) with linear probes (18 MHz and 9 MHz) and SONIMAGE HS-1 (Konica Minolta, Inc, Tokyo, Japan) with linear probes (18 MHz and 11 MHz). MRI equipment was a 3T unit (Discovery MR750W, GE Healthcare Technologies, Inc., Chicago, IL, USA).

Ethics approval was granted by the institutional clinical research ethics committee of Waseda University.

### 2.1. Ultrasound Diagnosis Procedure of Proximal Hamstring

**Figure 2:**
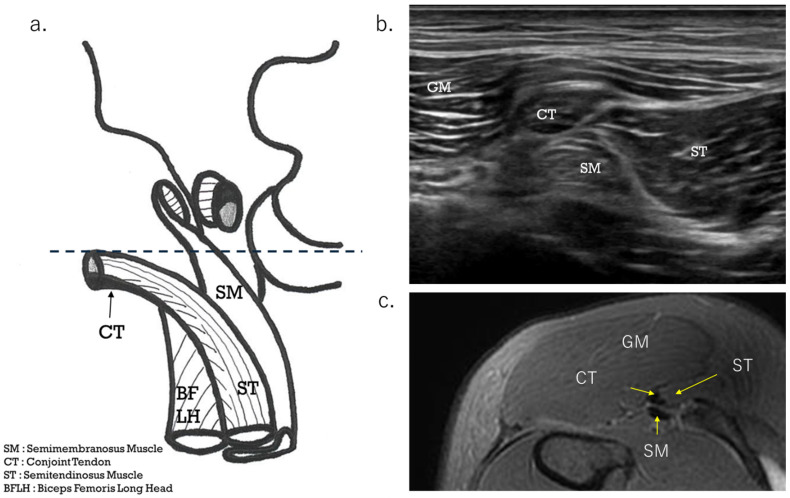
Short-axis view at the level of the CT and SM tendon cross. (**a**) Schema showing CT-ST and SMT flipped from the attachment point. The position shows the relationship between the CT and the SM tendon (SMT). When the ultrasound probe is scanned from the distal to the proximal end, the SMT travels from the medial side to the lateral side, through a deeper layer than the CT. As for the ischial tuberosity attachment point, the SMT is located at the lateral side, with the CT and ST muscle fibers located at the medial side. (**b**) Ultrasound short-axis image at the point where the CT and SMT intersect. The CT and SMT ellipses are lined up vertically. (**c**) This is an axial MRI image (T2-weighted image with fat suppression) of the same level.

The diagnostic procedure began with an ultrasound scan. If there was tenderness around the ischial tuberosity, the area in the short axis from a slightly more distal position was observed at the level where CT and SMT intersect (Figure 2a, dashed line). The severity of the injury to the CT tear was assessed by following the short-axis view from distal to proximal up to the ischial tuberosity. The avulsion or tear site was confirmed in the long-axis view. The distance from the tear to the ischial tuberosity was then clear. If there was tenderness around the proximal musculotendinous junction, it was observed whether any hematoma was present on both sides or only on one side on CT, and the uneven thickness of CT in the short axis was checked. The probe was then placed in the direction of the muscle fibers at the musculotendinous junction to determine the injury type and severity of damage to the tendons and muscle fibers (in the long-axis view). In cases where it was difficult to judge, the decision was made by comparing it with the healthy side.

### 2.2. Grades of Ultrasound Diagnosis

The initial diagnosis was made using ultrasound, and the final diagnosis was made using MRI. The diagnosis by ultrasound was evaluated in the following three grades.

0. No diagnosis was possible.
1. The existence of a muscle tear could be suspected.
2. The diagnosis of a muscle tear was certain.

We defined a positive US diagnosis as one that showed findings in grades 1 and 2. We then compared the probability of detection (sensitivity) with US to MRI.

## 3. Results

There were 52 male limbs, with a mean age of 21.5 years (18–23 years). In 40 cases, the BFLH-ST complex was damaged, and in 12 cases, the SM was damaged. The detailed damage distribution and the diagnostic details of each damaged area with ultrasound are shown in Table 1.

Damage to the BFLH-ST complex was observed in 40 cases (76.9%). Type I injuries, those CT tears that were of the ST and BFLH, were observed in 19 limbs (36.5%). Osteotendinous junction tears were observed in one limb, tendon tears in 14, and the MTJ type in 4. BFLH injuries without CT injury, i.e., Type II, were 19 cases (36.5%), which included 10 in the proximal musculotendinous junction (MTJ)—myofascial junction (MFJ) injuries, 5 intermuscular injuries, and 4 distal MFJ injuries. ST injuries without CT injury, i.e., Type III, consisted of 2 cases (3.8%), 1 proximal intermuscular injury and 1 distal intermuscular injury. In the image diagnosis of BFLH-ST complex muscle tears, the sensitivity of ultrasound diagnosis was low for injuries involving the MFJ of the BFLH.

Type SM injuries were observed in 12 cases (23.1%), including 1 OTJ injury, 5 proximal tendon tears, and 6 proximal MTJ tears. There was no BFSH tear. There were 4 cases in which two flesh injuries appeared at approximately the same time. The athlete complained of symptoms in the area of the major injury, but there was also a minor injury in another area. In terms of distribution, the area of the major injury was registered, and the details are shown in Table 2.

In the BF-ST complex, Type I, which is damage to the origin of the CT or damage to the tendon itself, was able to be detected in all but one case. In Type II, it was difficult to detect even slight damage to the MFJ. SM damage could be determined except for slight proximal MTJ damage. The number of injuries detected by ultrasound was compared with MRI diagnosis and shown as sensitivity of ultrasound (Table 3). The overall detection rate of ultrasound compared to MRI was 87%; Type II was low at 74%,Type I at 95% and Type III was detected in all cases.

FW players had significantly more SM injuries than BK players, and conversely, BK players had more BF-ST complex injuries.

## 4. Discussion

In sports injuries, proximal hamstring injuries are frequent and often problematic due to the time taken to return to play. In the present study, we have performed an anatomical classification of injury sites with a focus on the two ischial tuberosity attachments and investigated their frequency. The conjoint tendon (CT) of the hamstring muscle complex (HMC) is the origin of the BFLH-ST complex at the ischial tuberosity [3,6,10]. In other words, CT injury is an injury that involves both medial and lateral hamstring elements. BFLH-ST complex-related injuries accounted for the majority of hamstring complex injuries (40 cases, 76.9%). CT injuries were observed in 19 limbs (36.5% of the total) in Type I. By muscle, BFLH injuries (Types I and II) were found in 38 limbs (73.1% of the total). 19 limbs were CT injuries and 10 limbs had proximal MTJ injuries of BFLH; that is, 76.3% of the BFLH injuries were proximal injuries. ST injuries, Type I and Type III, numbered 21 (40.1% of the total). The raphe is located in the middle of the semitendinosus muscle, and all injuries proximal to the raphe included CT injuries. In other words, 90.1% of semitendinosus injuries were CT injuries. There were 12 cases of SM injury type. There was no case of BFSH injury. The reason for this is thought to be that it is a single-joint muscle.

In a 2016 study [5], Crema examined the distribution of hamstring tear locations in 275 cases, finding muscle tears at 393 sites, including 222 tears of the BFLH (80.7%), 96 of the ST (43.2%), 54 of the SM (24.3%), and 22 of the BFSH (9.9%). The results of this previous study largely match our distribution.

The area where the BFLH—the biarticular muscle responsible for external rotation of the lower limb—and the ST—the biarticular muscle responsible for internal rotation—meet is susceptible to mechanical stress. That is, clinically, it is important to evaluate CT injuries, as this can be useful not just for deciding initial surgical indications, but also for formulating rehabilitation programs and determining the timing of recovery [2,4,6,13].

In another paper, the authors investigated the location of hamstring injuries in professional rugby players and found that 73% of the injuries were to the BFLH, making it the most common location. They also reported that 53% of the BFLH injuries were to the distal myofascial junction [14]. In this study, too, damage to the distal side of the BFLH was more common in the myofascial area than at the musculotendinous junction.

### 4.1. Player’s Position

We divided the positions into forwards (FWs, positions No. 1–8) and backs (BKs, positions No. 9–15), and found that the BFLH-ST complex injuries were more likely to occur in FWs than in BKs; on the other hand, the SM injuries tended to be more common in FWs (Table 4). According to a previous paper [15], SM injuries are a type of stretch injury that is more likely to occur when the knee joint is extended while the hip joint is flexed. It is thought that many cases of hamstring injuries occur when FW players are in a position, such as tackle-like posture and ‘jackal’ posture for stealing the ball from the enemy.

### 4.2. Osteotendinous Junction/Proximal Tendon

**Figure 3:**
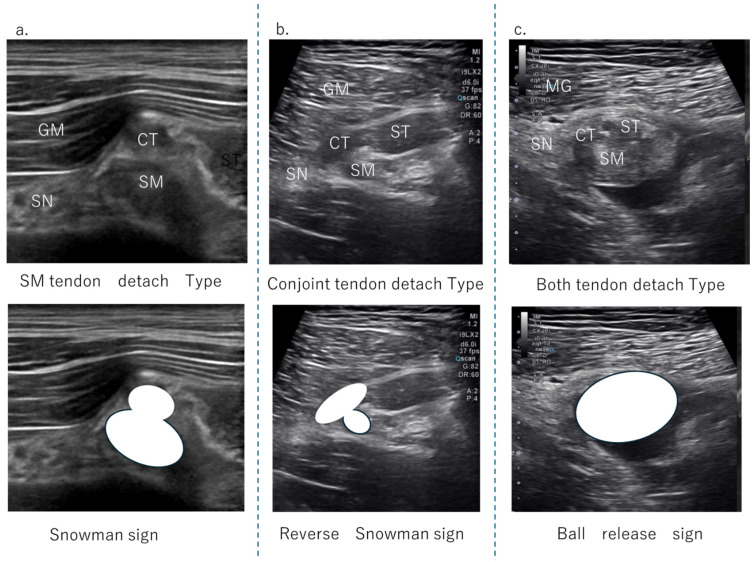
The US findings of the tendon detachment. (**a**) This is a case of SM detachment from the attachment point. Compared to CT, the SMT that exists in the deeper layers regresses, and the volume in this area often increases, resulting in an image like a ‘snowman sign’. (**b**) If there is a tear or damage to the CT, the surface layer will become a larger oval, presenting a ‘reverse snowman sign’ appearance. (**c**) When both the CT and SMT are torn apart as a whole, they are depicted as a single mass, or ‘ball release sign’. This expresses the appearance of the attachment area separating from the ischial tuberosity as a single mass, like a ball.

**Figure 4:**
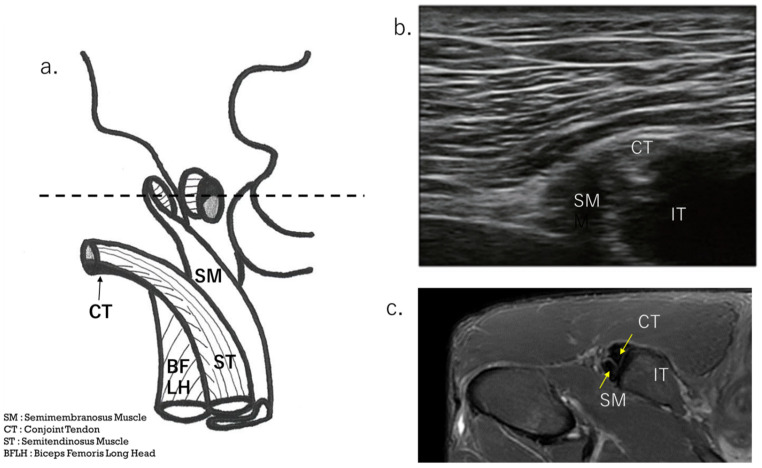
Short-axis view at the level osteotendinous junction at ischial tuberosity. (**a**) Schema showing the attachment of the ischial tuberosity; (**b**) normal ultrasound short-axis image of the ischial tuberosity attachment; (**c**) normal axial image (T2-weighted image with fat suppression) taken using MRI.

**Figure 5:**
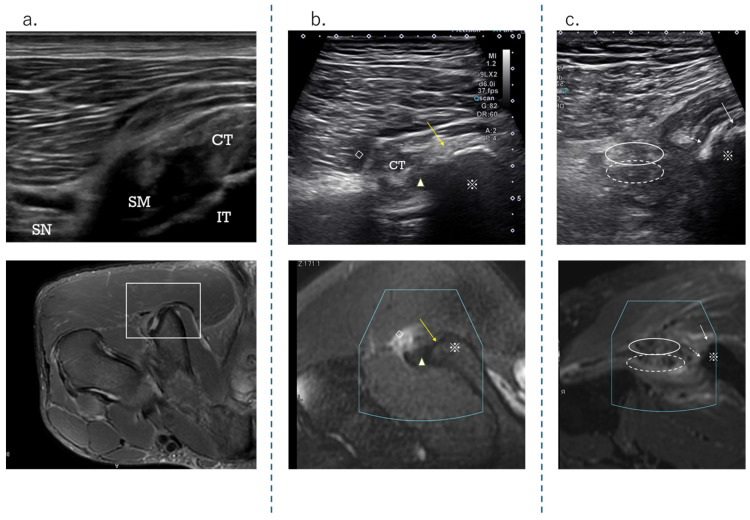
The US findings (upper row) and MRI images (lower row) of the originate tendon detachment at ischial tuberosity. (**a**) Detached image of the SM tendon attachment; (**b**) detached image of the CT attachment (yellow arrow), hematoma (white square), and SM attachment (solid white triangle); (**c**) detached image of the CT attachment (white arrow) and CT (white ellipse), detached SM attachment (white dashed arrow), and SMT (white dashed ellipse). 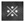: Ischial tuberosity.

The HMC is a biarticular muscle complex made up of four muscles, three of which originate from the ischial tuberosity with the CT and SMT. The ST is composed of muscle fibers that originate both directly from the ischium and from the conjoint tendon (CT), whereas all BFLH muscle fibers originate from the CT [2,3,8,10] (Figure 2a). Type I injuries are defined as a tear of the CT. As in the diagnostic procedure described above, by carefully observing via ultrasound, it is possible to determine the location of the damage. When performing an ultrasound evaluation of damage to the ischial tuberosity and surrounding area of the origin tendon, such as in CT and SMT, it is easier to see the detached and coiled appearance of the damaged tendon and find swelling if the evaluation is performed at the level where the CT and SMT intersect (Figure 2a, dashed line). The characteristics of the ultrasonic short-axis findings in cases of CT injury and SMT injury and injury to both the CT and SMT are shown in Figure 3. The position of the attachment points of the SMT, CT, and ST muscle fibers are important. The SMT runs through the deep layer of the CT and attaches to the lateral side of the ischial tuberosity. The CT attaches to the medial side of the SMT, and the muscle fibers of the ST attach further to the medial side. The area where the SMT attaches is difficult to visualize using ultrasound in athletes with well-developed gluteal muscles or those with a lot of subcutaneous fat. In such cases, it is useful to evaluate by scanning from the points in Figure 2 and Figure 3 towards the proximal end. The short-axis image of the ischial tuberosity is shown next, in contrast to the MRI image (Figure 4 and Figure 5). In this study, detection by ultrasound was possible for both the BF-ST complex and SM in all but one case, which was a minor injury without conjoint tendon tortuosity or hematoma.

### 4.3. Proximal Tendon—Musculo Tendinous Junction

**Figure 6:**
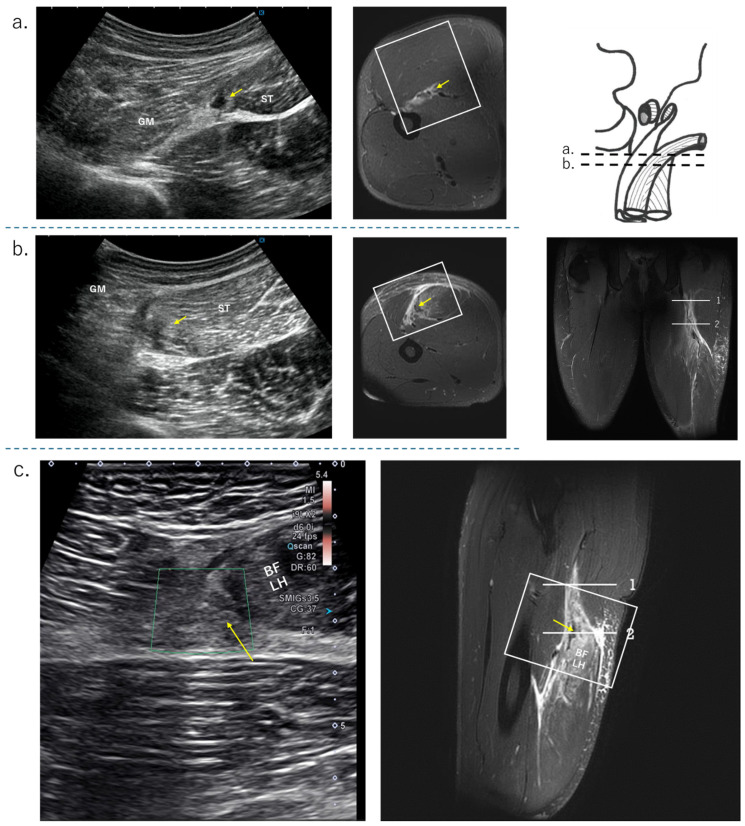
The US findings of the CT tear, MRI condition: T2-weighted image with fat suppression. (**a**) short-axis view at level 1, CT rapture and hematoma; (**b**) short-axis view at level 2, tortuosity CT (yellow arrow), and ST side hematomas, BFLH side hematomas; (**c**) long-axis view, CT dropped to the periphery (yellow arrow) due to CT rupture. Refer to the sagittal MRI image.

Injuries extending from the proximal CT of the ST and BFLH to the myotendinous junction are also very common [3,8]. A full-thickness injury to the free tendon of the BFLH will result in a hematoma on both the ST and BFLH sides. As the severity of the muscle tear increases, the tension at the periphery of the torn area decreases, and it becomes tortuous (Figure 6). In the case of an ultrasound short-axis image, if the surrounding hematoma in the CT is large, it can be detected, but if the hematoma is small, it is difficult to detect, and diagnosis is performed together with MRI. It is easy to detect those with torn CTs that had retracted distally in the long-axis view. In addition, for damage to the myotendinous junction, if the continuity is clearly lost, it can be determined in the long-axis view, but if the damage is slight, it is necessary to check with MRI. In this study, it was difficult to detect microscopic injuries without hematomas, especially those at the myofascial junction (MFJ), using ultrasonography.

### 4.4. Biceps Femoris Tear

**Figure 7:**
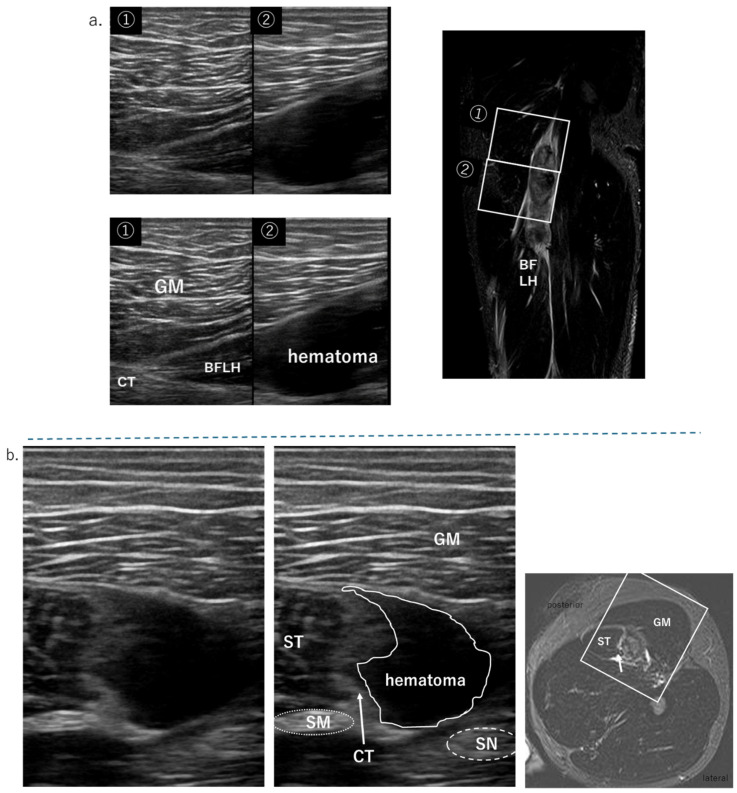
The US findings of proximal MTJ tear of BFLH. GM: gluteus maxim; SN: sciatic nerve, MRI condition: T2-weighted image with fat suppression; (**a**) long-axis view, no bending of the CT and hematoma on the BFLH side of the CT; refer to the coronal MRI image; (**b**) short-axis view, hematoma on the BFLH side of the CT (white arrow), but not on the ST side.

In this section, we will discuss injuries of the distal portion of the BFLH (Type II) and of the BFSH. The proximal MTJ of BFLH has a strong pennate structure. This part of the body is prone to muscle strain. Figure 7 shows an MTJ injury on the BFLH side of the CT. First, a hematoma is confirmed on the BFLH side of the CT in the short-axis image of the ultrasound, and the extent of the MTJ damage is confirmed in the long-axis image of the BFLH muscle fiber direction. A detailed evaluation of whether the damage has spread to the ST side of the CT is performed using MRI findings. There were three cases of slight damage to the fascia on the starting side of the MFJ, which was difficult to detect using US. There were five cases of intramuscular injury and injury to the aponeurosis within the muscle that is continuous with the distal fascia. There were also four cases of distal myofascial injury. The distal myofascial junction also has a pennate structure and is a common site for muscle tears. In this survey, there were no ruptures or avulsions of the distal tendon. Such slight MFJ injuries in BFLH often have no hematoma, are relatively deep, and may be difficult to delineate muscle fibers, resulting in low ultrasound detection (Table 3). The biceps femoris short head (BFSH) is a uniarticular muscle of the knee and is less frequently injured.

### 4.5. Semitendinosus Muscle Tear

**Figure 8:**
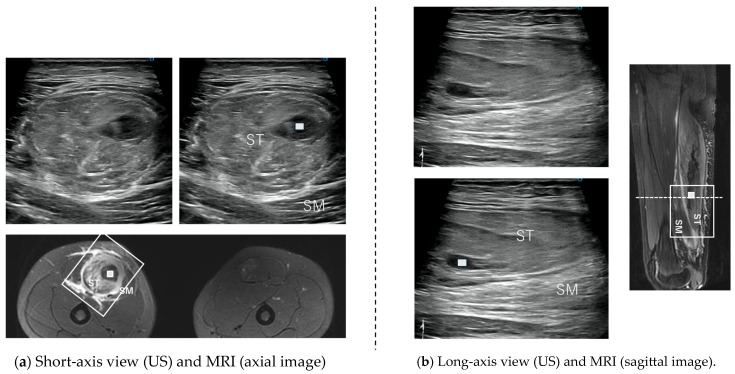
The US findings of a distal intermuscular tear of the ST/MRI condition: T2-weighted image with fat suppression; (**a**) there is a diffuse high-echo area of edema around the hematoma (white square). This section is distal to the raphe (dashed line in sagittal image); (**b**) there is a hematoma and diffuse edema due to the tear in the ST. There is SM in the deeper layers, and the boundary with the shallow ST is clear.

It is well known that the semitendinosus (ST) originates from the ischial tuberosity and shares the CT with BFLH [2,8,10]. ST injuries with CT tears are Type I and those without CT tears are Type III in our system. The origin of the ST is divided into an area where muscle fibers appear directly from the ischium and the musculotendinous junction where muscle fibers appear from the conjoint tendon [10,16]. Further, the raphe in the center of the muscle belly divides it into proximal and distal muscle bellies [12]. The muscle fibers are fusiform-shaped. Most of the ST injuries were proximal injuries, including the CT injuries. Type III of this classification was easily diagnosed on both the short and long axes of ultrasound, with MRI only as a reference. Intramuscular spatial resolution was better with ultrasound than with MRI. Figure 8 is a case of distal intermuscular injury distal to the raphe.

### 4.6. Semimembranosus Muscle Tear

**Figure 9:**
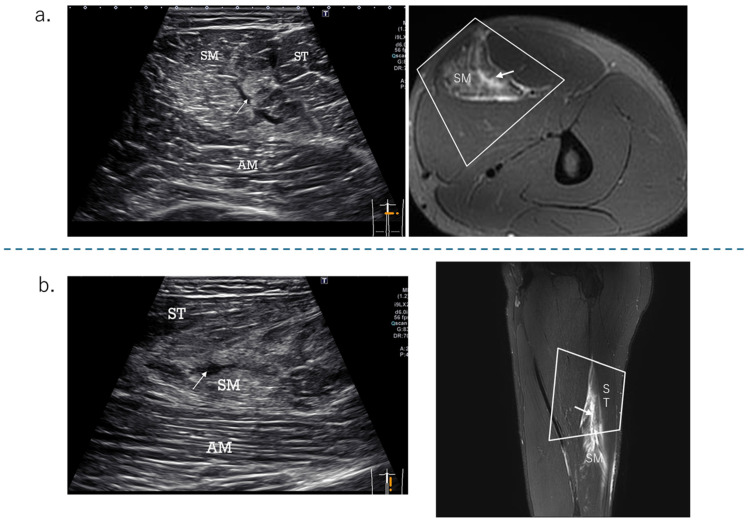
The US findings of the SM proximal musculotendinous junction tear. (**a**) Ultrasound short-axis image: a tear and hematoma (white arrow) are seen at the proximal MTJ of the SM. (**b**) Long-axis image: a tear and hematoma (white arrow) and edema are seen between the ST and AM (adductor magnus). It is easier to understand if you refer to the sagittal MRI image.

The proximal SM consists of a membranous tendon component, and the aponeurosis of its origin extends to the center of the femur [12]. The muscle fibers have a pinnate structure [9]. Figure 2, Figure 3, Figure 4 and Figure 5 are the osteotendinous junction injury. Figure 9 was a proximal MTJ tear of the SM, and the muscle fiber structure of this area is strongly pennate.

Ultrasonographic diagnosis of an injury to the originating tendon has been described above, but an MTJ injury can be easily determined by observing a short-axis image with swelling of the originating tendon and a hematoma in the distal muscle–tendon transition area. MRI is only for reference, and ultrasound alone can be used to make a diagnosis in MTJ injuries of the SM type. The findings of intramuscular muscle fiber tears and hematomas were clearer with ultrasound. All of the SM type injuries were related to the origin tendon or the myotendinous transition zone of the origin tendon.

### 4.7. Sonography and MRI

Sonography is quicker, more accessible, and less expensive than MRI [17,18,19]. In addition, using high-resolution sonographic equipment enables the detection of minute injuries. Connell et al. compared sonography and MRI for assessing hamstring injuries [17,18,19,20,21]. Sixty professional male football players presented with suspected acute hamstring strain and underwent sonography and MRI within 3 days of injury. HMCI was detected in 42 (70%) of 60 patients with MRI and in 45 (75%) patients with sonography. They described sonography as an extremely useful, low-cost tool for assessing acute hamstring injuries, which is roughly as accurate as MRI. In the present study as well, high-resolution sonography was useful in primary diagnosis, sufficient to diagnose all but the most subtle damage to the tendon and MFJ injuries. In particular, it was difficult to detect even slight damage to the MFJ in the BFLH. Compared to MRI, 45 of 52 cases could be diagnosed by ultrasound (Table 3). Ultrasound can detect disruptions in the alignment of muscle fibers. As shown in Figure 8 and Figure 9, the assessment of fine structural disruption of muscle fibers is comparable to or better than MRI.

On the other hand, MRI was useful for the qualitative evaluation of damage to tendon components such as the ischial tuberosity attachment. Ultrasound and MRI examinations are typical methods for evaluating images of muscle strains [2,16,18,22], but there are few initial medical institutions equipped with MRI. For this reason, we believe that the ideal method is to use US as the initial diagnosis and then use MRI to assist with that diagnosis. This article also explains the ultrasound diagnosis and local findings for each type of injury.

### 4.8. Other Classifications

Various muscle injury classification systems have been reported [6,7,23,24,25,26]. In 1995, Takebayashi reported a sonographic classification system, in which tears to ≤5% of muscle fibers met grade I criteria, partial tears of ≥5% were grade II, and complete tears were grade III [25]. In 2002, Peetron reported negative as grade 0, ill-defined hyper- or hypoechoic without objective muscle discontinuity as grade I, partial tear as grade II, and complete tear as grade III [26]. These are related to the evaluation of muscle damage in ultrasound examinations. In 2013, Muller-Wohlfahrt [24] added spine-related neuromuscular disorders and muscle-related neuromuscular disorders to fatigue-induced and delayed-onset muscle soreness for direct and indirect muscle injuries from direct external force but without ultrasound findings. In 2014, Pollock et al. [7] reported a British athlete muscle injury classification, which classifies injuries by site as myofascial, musculotendinous, and intratendinous, and by severity with grades ranging from 0–4. In 2018, Valle proposed a hamstring injury classification that included parameters such as the injury mechanism, site, severity [6]. Valle’s classification divides the injured area into the proximal, middle, and distal parts, but there is no classification for CT injury.

None of these classifications allow for evaluating the structure of the proximal hamstring anatomy such as the BFLH-ST complex. However, hamstring muscle tears around the ischial tuberosity often take a long time to heal, which can be a problem. Our classification system includes categories that reflect anatomical structure, involving avulsion from the ischial tuberosity and proximal tendon tears. We believe that assessing the discontinuity of the proximal portion of the tendon is useful for determining whether to advise surgery or conservative therapy. 

In this study, ultrasound was found to be useful in the primary diagnosis of originating tendon injuries. First, the separation of the flesh should be detected in ultrasonography, and detailed evaluation of the bone attachments and tendon tissue should preferably be done with MRI. Further, it is considered important to evaluate the severity of muscle tears within 48 h of injury. Even if MRI is not available, the primary provider can perform the initial evaluation with sonography. If a Type I CT injury or proximal tendon tear is found, a decision can be made as to the necessity and urgency of MRI.

### 4.9. Limitations

The number of cases is small and the subject has been limited to male university rugby players. This study is a prospective clinical survey, and the reliability of ultrasound examinations has not been evaluated.

In the future, as a development of this study, we would like to increase the number of cases and carry out research on the relationship between the players position and the anatomical site of injury, as well as the relationship between the anatomical site of injury and the time it takes to recover.

## 5. Conclusions

We performed an ultrasound-based imaging study of a hamstring tear in university rugby players. We investigated the frequency of occurrence of injury sites classified by the tendon of origin and presented the ultrasound findings. MRI was necessary for qualitative assessment of tendon injuries, but otherwise, the diagnosis could be made down to the site and extent of injury by ultrasound. We are pleased to report that ultrasonography was found to be sufficient to make an initial diagnosis (i.e., as a detection). We were particularly interested to find that injuries related to the originating tendon were highly prevalent. We are planning to continue with our research and to look at the return-to-play times for each type of injury.

## Figures and Tables

**Table 1:** Distribution of injury location and detection grade of ultrasound.

Injury Location	Distribution (N)	Ultrasound Detection Grade		
	(MRI Diagnosis)	0	1	2
**BFLH-ST complex injury**	**40**	**6**	**19**	**15**
Type I (CT tear)	19	1	10	8
Osteotendinous junction (OTJ)	1		1	
Tendon	14	1	6	7
MTJ	4	0	3	2
Type II (BFLH injury without CT tear)	19	5	8	6
Proximal MTJ—MFJ	10	3	4	3
Intermuscular	5	0	3	2
Distal MFJ	4	2	1	1
Distal MTJ/Tendon/OTJ				
Type III (ST injury without CT tear)	2		1	1
Proximal MTJ				
Proximal intermuscular	1		1	
Distal intermuscular	1			1
Distal MTJ/Tendon/OTJ				
**SM injury**	**12**	**1**	**6**	**5**
OTJ	1			1
Proximal tendon—MTJ	5		3	2
Proximal MTJ—intermuscular	6	1	3	2
Distal MTJ/Tendon/OTJ	0			
**BFSH injury**	0			
**Total**	**52**	**7**	**25**	**20**

**Table 2:** Four cases of simultaneous muscle tears in two location.

Major Injury	Minor Injury	Number
Type I tendon tear	SM proximal MTJ injury	2
SM proximal MTJ injury	Type II distal MFJ injury	2

**Table 3:** Ultrasound sensitivity compared with MRI diagnosis at each injury location.

Injury Location	US Positive	MRI Diagnosis	US Sensitivity
**BFLH-ST complex injury**	**34**	**40**	**0.85**
Type I: (CT tear)	18	19	0.95
Type II: (BFLH injury without CT tear)	14	19	0.74
Type III: (ST injury without CT tear)	2	2	1.0
**SM injury**	**11**	**12**	**0.92**
**BFSH injury**			
**Total**	**45**	**52**	**0.87**

**Table 4:** Injury type and player’s position.

	Forward Player	Backs Player	Total
BFLH-ST complex injury	15	25	40
SM injury	8	4	12

BFLH: biceps femoris long head; ST: semitendinosus; SM: semimembranosus.

## Data Availability

The data presented in this study are available on request from the corresponding author (M.W.). The data are not publicly available due to privacy concerns.
